# Resistance Change Mechanism of Electronic Component Mounting through Contact Pressure Using Elastic Adhesive

**DOI:** 10.3390/mi10060396

**Published:** 2019-06-14

**Authors:** Takashi Sato, Tomoya Koshi, Eiji Iwase

**Affiliations:** 1 Department of Applied Mechanics, Waseda University, 3-4-1 Okubo, Shinjuku-ku, Tokyo 169-8555, Japan; sato@iwaselab.amech.waseda.ac.jp; 2 Sensing System Research Center, National Institute of Advanced Industrial Science and Technology (AIST), 1-1-1 Higashi, Tsukuba 305-8565, Japan; t.koshi@aist.go.jp

**Keywords:** surface mounting, flexible electronic device, contact resistance, contact pressure

## Abstract

For mounting electronic components through contact pressure using elastic adhesives, a high contact resistance is an inevitable issue in achieving solderless wiring in a low-temperature and low-cost process. To decrease the contact resistance, we investigated the resistance change mechanism by measuring the contact resistance with various contact pressures and copper layer thicknesses. The contact resistivity decreased to 4.2 × 10^−8^ Ω·m^2^ as the contact pressure increased to 800 kPa and the copper layer thickness decreased to 5 µm. In addition, we measured the change in the total resistance with various copper layer thicknesses, including the contact and wiring resistance, and obtained the minimum combined resistance of 123 mΩ with a copper-layer thickness of 30 µm using our mounting method. In this measurement, a low contact resistance was obtained with a 5-µm-thick copper layer and a contact pressure of 200 kPa or more; however, there is a trade-off with respect to the copper layer thickness in obtaining the minimum combined resistance because of the increasing wiring resistance. Subsequently, based on these measurements, we developed a sandwich structure to decrease the contact resistance, and a contact resistivity of 8.0 × 10^−8^ Ω·m^2^ was obtained with the proposed structure.

## 1. Introduction

Recently, flexible electronic devices, such as flexible displays [1,2,3,4,5], batteries [6,7,8,9,10], and sensor arrays [11,12,13,14,15], have been developed by many research groups [16,17,18,19,20,21,22]. In particular, healthcare monitoring systems using flexible electronic devices, which can adhere to human skin, have attracted considerable interest. In previous studies, to mount electronic components on a flexible circuit, solders [23,24] or conductive adhesives [25,26] were used, and the contact resistivity is on the order of 10^−11^ Ω·m^2^ in solders and 10^−9^ Ω·m^2^ in conductive adhesives. The conductivity is the contact resistance per unit contact area. Although a simple fabrication process is required for high-mix, low-volume manufacturing to achieve individual optimization of healthcare monitoring systems, these fabrication processes are quite complicated owing to both the dispensing and patterning processes. Moreover, polyurethane and rubbers are mainly used for substrates of stretchable circuits, but their low heat resistance causes problems in soldering and misalignment of electronic components owing to thermal expansion. To solve these issues, solderless mounting methods via contact pressure using an elastic adhesive [27,28] were proposed as a new simple manufacturing processes. These methods do not require complex mounting processes and heating. Their applicability is, however, hindered by their high contact resistance because the electrical connections are based on physical contact. The mechanism for controlling resistance change to achieve low contact resistance is still not clear.

Therefore, in this study, we first considered the mechanism of contact resistance change to clarify the causes of this change. Then, the change in the contact resistance was experimentally measured to investigate the relationship between the expected causes and the contact resistance. Second, we developed a new mounting structure to obtain a high compression force to decrease the contact resistance and evaluated the contact resistance.

## 2. Materials and Methods

**Figure 1:**
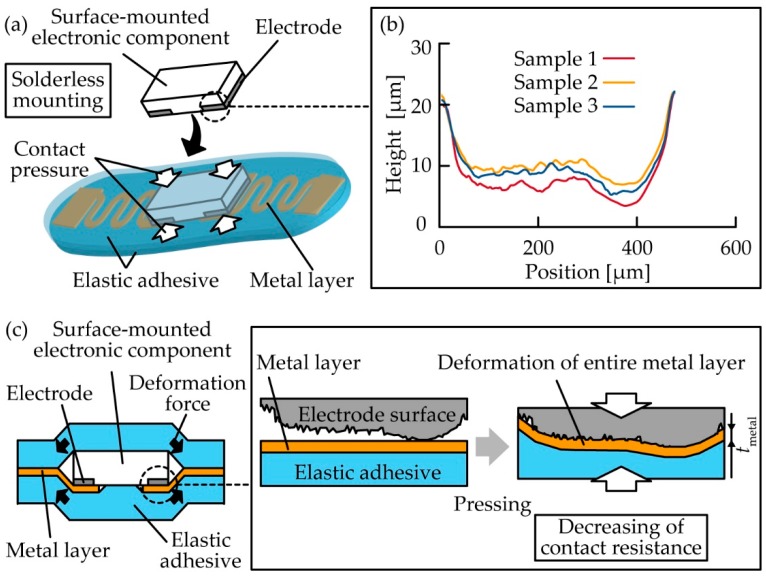
(**a**) Schematic image of electronic component mounting through contact pressure using an elastic adhesive; (**b**) electrode surface profiles of surface-mounted electronic component; (**c**) deformation of contact pad of metal layer of elastic adhesive sheet by gradually increasing contact pressure.

First, the basic mechanism of contact resistance was considered. Contact resistance is the sum of the constriction resistance and film resistance. The constriction resistance is influenced by the concentration of current that flows into the real contact area between the conductor surfaces. The film resistance is influenced by chemical films, such as oxide films, oil, and dirt films, on the conductor surface. In this research, we focused on constriction resistance because the constriction resistance must be dominant in this solderless mounting of electronic components; constriction resistance would decrease as the real contact area increased. Figure 1a shows the schematic image of the contact resistance change in the solderless mounting method. The electrical connection is formed via contact of surface-mounted electronic components with contact pads of the metal layer, where the elastic adhesives provide restoring forces to press them. For example, a surface-mounted electronic component is held by elastic adhesives sandwiching from above and below in the sandwich structure proposed in this research, shown in Figure 1c. The important feature here is the surface shape of the electrode of the electronic components, as shown in Figure 1b. Most of the electrodes in surface-mounted electronic components are generally not perfectly flat but slightly curved and rough, and so are the chip resistors. These electrodes are gradual convex shapes 400-µm wide and 20-µm high, and the surface roughness of the electrodes is less than a few micrometers. Figure 1 shows the decrease in the contact resistance caused by the change in the contact area when an electronic component is pressed against the contact pads. On applying the contact pressure, the contact pad deforms and the contact area with the electrodes of the component is enlarged. Hence, Figure 1c suggests that the thinner metal electrodes exhibit large deformation, and thus they make contact with the electrodes to lower the contact resistance. Nevertheless, the thinner metal layers show larger resistance and are susceptible to damage owing to excessive deformation. Therefore, it is important to investigate contact pressures to obtain a sufficiently small contact resistance with different metal layer thicknesses and determine the thickness when the sum of the contact resistance and wiring resistance is the lowest.

**Figure 2:**
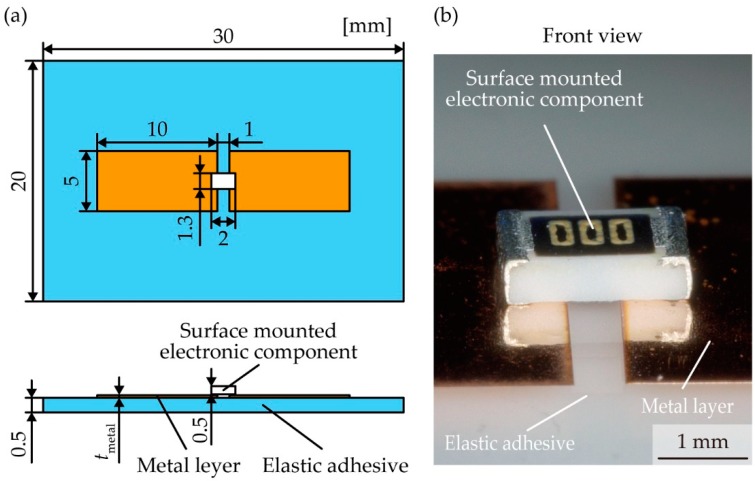
(**a**) Schematic images of experimental sample; (**b**) optical image of sample.

**Figure 3:**
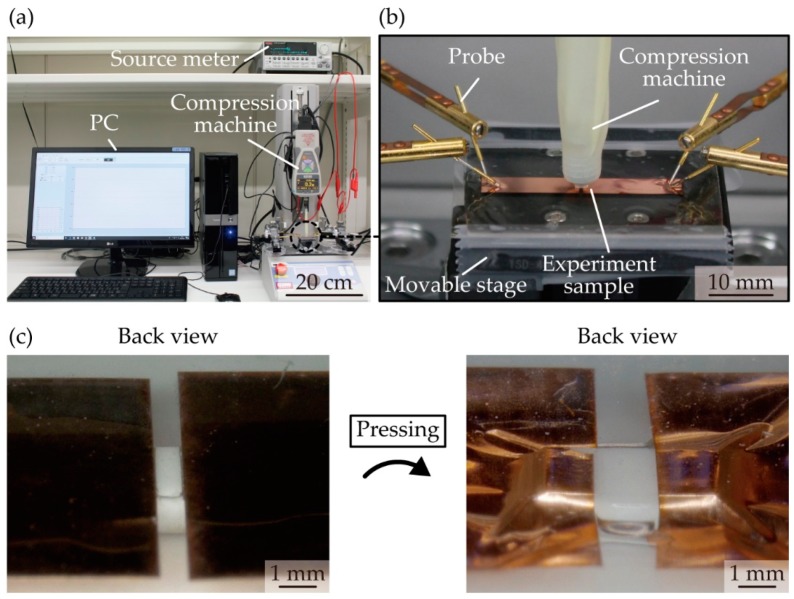
Optical images of experimental apparatus used to measure the change in contact resistance. (**a**) Optical image of apparatus; (**b**) optical image of enlarged view of experimental sample pressed by compression testing machine; (**c**) optical images of experimental sample with and without pushing.

**Figure 4:**
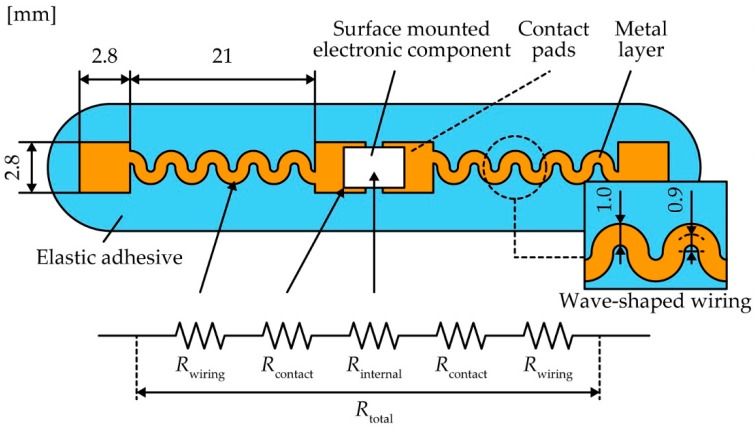
Schematic image of sample device.

To evaluate the effect of the contact pressure and thickness of the metal layer on the contact resistance, we experimentally measured the change in the contact resistance considering the contact pressure and thickness of the metal layer. Figure 2a shows schematic images with the dimensions of the experimental sample. The acrylic foam double sided adhesive sheet (Y-4905J, 3M, Maplewood, MN, USA) with a thickness of 0.5 mm was cut to a length of approximately 30 mm and width of 20 mm and affixed to a glass plate. A metal layer was patterned by laser cutting to a length of 10 mm and width of 5 mm using rolled copper films (Nilaco Co., Tokyo, Japan) with thicknesses of 5, 10, 30, and 50 µm. The metal layers were transferred onto the elastic adhesive with a gap of 1 mm, and a surface-mounted chip resistor (MCR10EZPJ000, ROHM Co., Kyoto, Japan) was placed on it. Figure 2b shows optical images of a sample used in the experiment. Figure 3a,b shows optical images of the apparatus used to measure the change in the contact resistance. The sample was fixed onto a movable stage with polyimide tape to be pushed by a compression testing machine (ZTS-20N, IMADA Co., Aichi, Japan); this machine has a pole with a small and flat end to press the chip resistor. The movable stage went up to apply pressure on the chip resistor, and the value of the pressure was monitored by the compression testing machine. The contact resistance was measured using a source meter (2614B, Keithley Instruments, Cleveland, OH, USA) with four-terminal sensing. The applied voltage was 10 mV, and the current compliance was 100 mA. Figure 3c shows the backside images of the experimental sample without and with pushing by a compression testing machine. With pressing pressure, the chip resistor sunk into the contact pad regions of the metal layer, and the contact pads largely deformed along the surface of the chip resistor. To measure and compare the total resistance, contact resistance (*R*_contact_), and wiring resistance of the metal layer (*R*_wiring_), we fabricated a sample device composed of a surface-mounted electronic component with a metal layer of contact pads and wave-shaped wirings and an elastic adhesive (Figure 4). We employed a chip resistor (MCR10EZPJ000, ROHM Co., Kyoto, Japan) with an internal resistance (*R*_internal_) of 11 mΩ, a 0.5-mm-thick acrylic foam adhesive sheet, and a metal layer of rolled copper films with thicknesses of 5, 10, 30, and 50 µm for the device fabrication. The copper films were patterned to form contact pads and wave-shaped wirings with a width of 1.0 mm and radius of 0.9 mm. The contact pads were fabricated with a length of 2.8 mm and width of 2.8 mm at both ends of the wiring. The patterned copper film was placed on the adhesive sheet with a gap of 1 mm. *R*_wiring_ of the wave-shaped electrodes was measured by the source meter with four-terminal sensing. Total resistance (*R*_total_) is the sum of *R*_contact_, *R*_wiring_, and *R*_internal_.

Based on the results obtained by measuring the above parameters, we fabricated several types of electrode structures to obtain lower resistance using higher pressure and investigated the relationship between the structures and compression force. We fabricated several samples of the simple adhesive, concave, and sandwich structures and compared the contact resistivity of each structure. In this measurement, we used an elastic adhesive with a thickness of 0.5 mm and a metal layer of rolled copper films with a thickness of 5 µm for device fabrication. The depth of the concave region was 0.5 mm because we used two elastic adhesive sheets to make the concave structure. The metal layers were patterned to the contact pad with a length of 10 mm and width of 5 mm formed via laser cutting. Then, the electrodes were transferred onto the elastic adhesive with a gap of 1 mm, and a 0.5-mm-thick chip resistor (MCR10EZPJ000, ROHM Co., Kyoto, Japan) as an electrical component was placed on it. The contact resistivity (*r*_contact_) was measured using the source meter 10 min after connecting. In addition, we measured the contact resistivity in the sandwich structure with other surface-mounted electronic components to investigate the encapsulation of the sandwich structure. A 0.6-mm-thick chip resistor (RK73ZW2HTTE, KOA CORPORATION, Tokyo, Japan), a 0.64-mm-thick chip resistor (3522ZR, TE Connectivity Ltd., Kanagawa, Japan), and a 1.1-mm-thick chip resistor (WSL251200000ZEA9, Vishay Intertechnology, Inc., Pennsylvania, USA) were mounted in a sandwich structure in the same manner as the chip resistor (MCR10EZPJ000, ROHM Co., Kyoto, Japan). Finally, to demonstrate the application of the sandwich structure for flexible electrical circuits, we fabricated an electronic device. A surface-mounted light emitting diode (LED) chip (OSR50805C1C, OptoSupply, N.T., Hong Kong, China) was embedded in the sandwich structure using a 5-µm-thick copper film and 0.5-mm-thick acrylic foam adhesive sheets to form an electrical contact.

## 3. Results and Discussion

**Figure 5:**
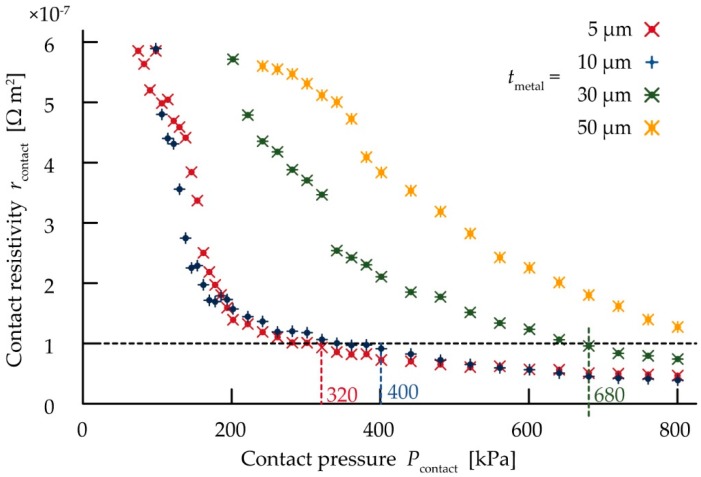
Relationship between contact pressure and contact resistivity with various copper layer thicknesses. The number of trials in each thickness was five.

**Figure 6:**
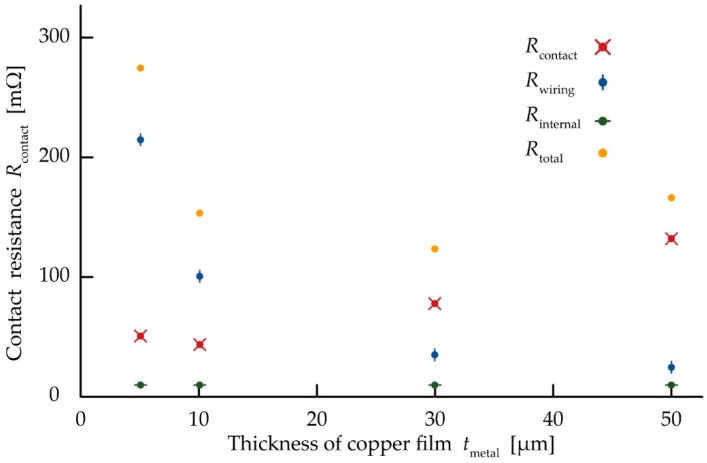
Relationship between thicknesses of copper layer *t*_metal_, contact resistance *R*_contact_, wiring resistance *R*_wiring_, internal resistance *R*_internal_, and total resistance *R*_total_. Five trials were carried out to measure *R*_contact_ for each thickness. Two trials were carried out to measure *R*_wiring_ and *R*_internal_ for each thickness.

Figure 5 shows the change in the contact resistivity (*r*_contact_) against various contact pressures (*P*_contact_) with various thicknesses of the copper contact pads (*t*_metal_). *r*_contact_ is the contact resistance per unit contact area. *r*_contact_ was much higher than 10^−2^ Ω·m^2^ at 0 kPa; it then dramatically decreased to 10^−7^ Ω·m^2^ as *P*_contact_ increased for each *t*_metal_. Then, *r*_contact_ gradually decreased to as low as approximately 10^−8^ Ω·m^2^ after *P*_contact_ increased. Moreover, the contact pressure values with an *r*_contact_ of 1.0 × 10^−7^ Ω·m^2^ for each *t*_metal_ are shown in Figure 5. The pressure value for an *r*_contact_ of 1.0 × 10^−7^ Ω·m^2^ decreased as *t*_metal_ decreased; these results indicate that the thin electrode decreases the pressure value at an *r*_contact_ of 1.0 × 10^−7^ Ω·m^2^. The sample device fabricated to measure and compare the total resistance, contact resistance (*R*_contact_), and wiring resistance of metal layer (*R*_wiring_) is shown in Figure 6. The copper films patterned to form contact pads and wave-shaped wirings are shown in Figure 4. *R*_contact_ at a pressure of 800 kPa was measured as shown in Figure 3. Figure 6 shows *R*_contact_, *R*_wiring_, *R*_internal_, and *R*_total_ for various values of *t*_metal_. *R*_contact_ decreased from 131.5 to 49.6 mΩ as *t*_metal_ decreased from 50 to 5 µm. *R*_wiring_ increased from 23.5 to 215 mΩ as *t*_metal_ decreased. *R*_total_ decreased as *t*_metal_ decreased from 50 to 30 µm, and the resistance reached a minimum value of 123 mΩ at a *t*_metal_ value of 30 µm. *R*_total_ remained the same as *t*_metal_ decreased from 30 to 10 µm, and then increased to 275 mΩ as *t*_metal_ decreased from 10 to 5 µm. Therefore, with these dimensions of the device, *R*_total_ reached a minimum value in the *t*_metal_ range 30–10 µm. This result indicates a trade-off point with respect to *t*_metal_ for minimizing *R*_total_.

**Figure 7:**
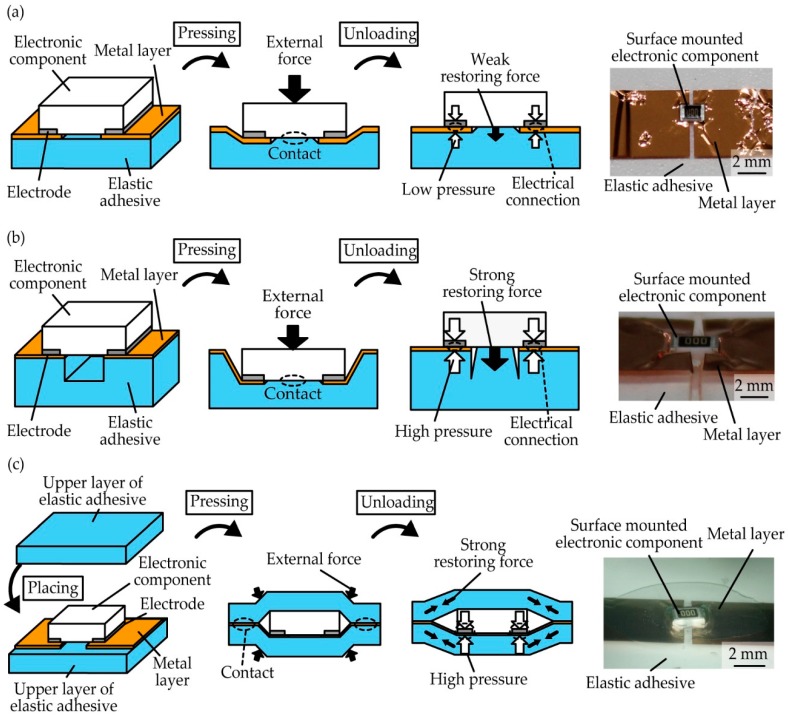
Schematic images and optical images of the (**a**) simple adhesive structure, (**b**) concave structure, and (**c**) sandwich structure.

Figure 7 shows schematics and optical images of the structures based on three different designs with the considered contact pressure and metal layer thickness for electrical connection from the above measurement results. In this measurement, we used an elastic adhesive with a thickness of 0.5 mm, a metal layer of rolled copper films with a thickness of 5 µm, and a surface-mounted electronic component with a height of 0.5 mm for device fabrication. Figure 7a is a simple adhesive structure, in which a surface-mounted electronic component is simply placed on the contact pads of the metal layer on the elastic adhesive. In this design, first, the electronic component is pressed against the contact pads through an external force, and the electronic component contacts the elastic adhesive. Then, after removing the external force, the elastic adhesive under the electronic component is deformed, and the restoring force of the deformed elastic adhesive presses the electronic component against the contact pads. As a result, an electrical connection is caused because of the restoring force. The restoring force based on the deformation of the elastic adhesive is weak, however, and the contact resistance is expected to be high. Therefore, we proposed a concave structure and a sandwich structure to increase the restoring force and obtain lower contact resistance in different ways. Figure 7b shows schematic images and an optical image of a concave structure, which is used with the non-uniform thickness substrate of the elastic adhesive. Owing to the concave shape of the elastic adhesive under the surface-mounted electronic component, the bottom of the concave shape of the elastic adhesive is stretched more, and the electronic component is pulled down stronger than in the simple structure. Figure 7c shows schematic images and an optical image of a sandwich structure. A surface-mounted electronic component is placed on the contact pads on a base layer of the elastic adhesive; then, an upper layer containing the elastic adhesive is placed on the electronic component and pressed via an external force to contact the base layer strongly. After removing the external force, the elastic adhesives are stretched to press the electronic component against the contact pads by providing a restoring force.

**Figure 8:**
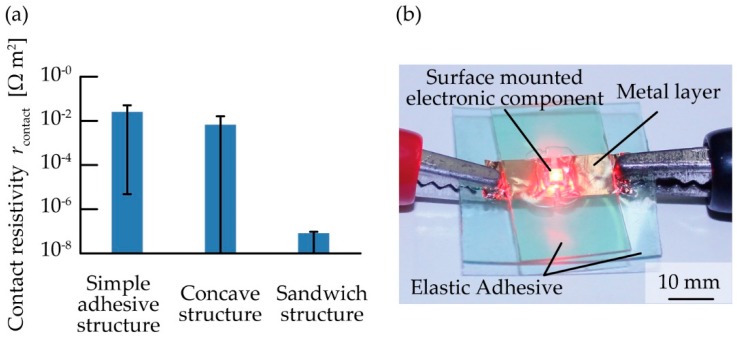
(**a**) Comparison of contact resistivity values in simple adhesive, concave, and sandwich structures; (**b**) flexible electronic device with light emitting diode (LED) chip mounted on a sandwich structure.

Further, we fabricated testing structures and obtained contact resistivity (*r*_contact_) values of 2.5 × 10^−2^, 1.9 × 10^−2^, and 8.0 × 10^−8^ Ω·m^2^ for the simple adhesive, concave, and sandwich structures, respectively, as shown in Figure 8a. Though the contact pressure value in these structures cannot be directly measured because the contact pressure is an internal pressure, we can estimate the contact pressure value from the contact resistance value based on the relationship between the contact pressure and the contact resistance explained in Figure 5. According to the values of the contact resistance in Figure 8a, the values of the contact pressure were estimated at less than approximately 60 kPa for the simple adhesive and concave structures, and more than approximately 320 kPa for the sandwich structure. The contact resistivity in the sandwich structure was on the order of 10^6^ Ω·m^2^ lower than that in the simple adhesive structure. This result indicates that the contact pressure was not sufficient to obtain low contact resistivity with the simple adhesive structures or concave structure. In the case of the concave structure, though high contact pressure was expected, it was considered that the elastic adhesive under the metal layer was deformed via tilting, and the contact pressure was decreased. The high contact pressure is also considered to be able to prevent the electrical component from slipping off the contact pads of the metal layer when a flexible electronic device using contact pressure is stretched or bent. Therefore, we are considering that the contact pressure is important for not only the contact resistance but also the mechanical stability. In addition, we confirmed that the contact resistivity in electronic components with different heights between 0.5 and 1.1 mm mounted by the sandwich structure was less than 5 × 10^−7^ Ω·m^2^. Therefore, our method can use electronic components with different heights. Figure 8b shows that an LED device fabricated using a sandwich structure can function as an electronic device. In this experiment, the light intensity of the LED device was not affected by the acrylic foam adhesive sheet because of the high transparency. We confirmed that the chip LED continued emitting light for ten hours. Because the chip LED seemed to keep almost same brightness ten hours later, we could consider that the contact resistivity of the chip LED was kept on the order of 10^−8^ Ω·m^2^ for more than ten hours. These results indicate that a low contact resistance can be obtained using the proposed sandwich structure, which facilitates the development of flexible electrical circuits through a simple and low-cost process.

## 4. Conclusions

We achieved a sufficiently small contact resistivity of 8.0 × 10^−8^ Ω·m^2^ between a surface-mounted electronic component and a flexible circuit substrate through pressure using an elastic adhesive. First, we investigated the mechanism of contact resistance change to obtain a low contact resistance. The change in contact resistance was measured at various pressures and using copper films having thicknesses of 5, 10, 30, and 50 µm. As a result, the contact resistivity decreased to below 10^−2^ Ω·m^2^ when the pressure increased to approximately 200 kPa. Above 200 kPa, the contact resistivity gradually decreased to 10^−8^ Ω·m^2^ as the contact pressure increased. Based on these measurement results, we designed a sandwich structure to obtain a resistivity of 8.0 × 10^−8^ Ω·m^2^. Moreover, we fabricated a simple flexible electronic device with an chip LED using the sandwich structure, and the chip LED continued emitting light for ten hours after mounting.
